# The impact of calcitriol and estradiol on the SARS-CoV-2 biological activity: a molecular modeling approach

**DOI:** 10.1038/s41598-022-04778-y

**Published:** 2022-01-13

**Authors:** Alireza Mansouri, Rasoul Kowsar, Mostafa Zakariazadeh, Hassan Hakimi, Akio Miyamoto

**Affiliations:** 1 Global Agromedicine Research Center (GAMRC), Obihiro University of Agriculture and Veterinary Medicine, Obihiro, Hokkaido Japan; 2 Department of Animal Sciences, College of Agriculture, Isfahan University of Technology, Isfahan, Iran; 3 Department of Biology, Payame Noor University, PO BOX 19395-3697, Tehran, Iran; 4 Research Institute of Bioscience and Biotechnology, University of Tabriz, Tabriz, Iran; 5 National Research Center for Protozoan Diseases, Obihiro University of Agriculture and Veterinary Medicine, Obihiro, Hokkaido Japan; 6 Department of Veterinary Pathobiology, College of Veterinary Medicine, Texas A&M University, College Station, TX USA

**Keywords:** Computational biophysics, Molecular modelling

## Abstract

The novel coronavirus disease (COVID-19) is currently a big concern around the world. Recent reports show that the disease severity and mortality of COVID-19 infected patients may vary from gender to gender with a very high risk of death for seniors. In addition, some steroid structures have been reported to affect coronavirus, SARS-CoV-2, function and activity. The entry of SARS-CoV-2 into host cells depends on the binding of coronavirus spike protein to angiotensin converting enzyme-2 (ACE2). Viral main protease is essential for the replication of SARS-CoV-2. It was hypothesized that steroid molecules (e.g., estradiol, progesterone, testosterone, dexamethasone, hydrocortisone, prednisone and calcitriol) could occupy the active site of the protease and could alter the interaction of spike protein with ACE2. Computational data showed that estradiol interacted more strongly with the main protease active site. In the presence of calcitriol, the binding energy of the spike protein to ACE2 was increased, and transferring Apo to Locked S conformer of spike trimer was facilitated. Together, the interaction between spike protein and ACE2 can be disrupted by calcitriol. Potential use of estradiol and calcitriol to reduce virus invasion and replication needs clinical investigation.

## Introduction

The novel coronavirus disease (COVID-19) pandemic, caused by the severe acute respiratory syndrome coronavirus 2 (SARS-CoV-2), was first recognized in the Hubei Province of China in December 2019 and has been reported as a very widespread disease with people-to-people transmission. Clinical evidence suggests that women are more resilient than men in terms of COVID-19^1–5^. In the previous study, the critical factors involved in increasing the mortality and severity of COVID-19 in patients were investigated and higher disease severity and mortality were found in male patients^2^. It has been reported that 12.8% of 86 men died and 75.6% recovered, while 7.3% of 82 females died and 86.6% recovered^6^. In addition, the reports showed a relatively low risk of incidence in children but a very high risk of death in seniors^7^. Elderly patients diagnosed with COVID-19 aged 60 or older^8^ had higher clinical signs, higher severity and longer periods of illness^9^.

Sex steroid hormones are the primary cause of female and male differences. Testosterone (T) known as the predominant sex steroid hormone in males plays an essential role in sexual and reproductive development. In women, the predominant sex steroid hormones progesterone (P4) and estradiol (E2) are produced by ovaries. With respect to the menstrual cycle, the concentration of E2 reaches the highest level just before ovulation (during the follicular or proliferative phase) and then decreases shortly afterwards (during the luteal or secretory phase). P4 is released at peak level during the luteal phase, and then drops before the next menstrual period. Decreases in menopausal-associated ovarian hormones have been well established^10^. Women with the lowest concentrations of androgen and E2 showed worse quality of life scores^11^. Steroids, such as E2, P4, T, and dexamethasone (DEX), may be involved in inflammation and immune reactions^12–15^. The expression of pro- and anti-inflammatory cytokines has been shown to change in the presence of steroid hormones^12–15^. Bianchi^13^ found that a low level of T is involved in the production and regulation of pro-inflammatory cytokines. Hormone P4 has been shown to weaken sepsis syndrome by suppressing the production of inflammatory cytokines such as IL-6 and TNF-α^14^. The efficacy of corticosteroids, such as DEX, has been reported for the reduction of pro-inflammatory mediators^16^. Corticosteroids can reduce mortality, need for mechanical ventilation, duration of mechanical ventilation, duration of ICU stay, and length of hospitalization for COVID-19 patients^17^. In addition, hydrocortisone (H) and prednisone (P) as alternative DEX candidates have been used to treat COVID-19 patients when DEX is not available^18–22^. Also, the effect of vitamin D on the treatment of COVID-19 has been reported recently^23–25^. Therefore, these findings clearly indicate the potential role of steroids in the control of COVID-19 infection. Moreover, due to their hydrophobic surface, steroids favor non-covalent interactions with a wide range of biomolecules, especially uncharged and aromatic amino acids^26–28^. As a result, steroids can strongly bind to proteins through hydrophobic interactions^26,27^.

Interaction between SARS-CoV-2 spike trimer glycoprotein and angiotensin converting enzyme-2 (ACE2) is widely recognized as a key step in coronavirus infection^29–31^. ACE2, an enzyme on the outer surface of the cells, plays a pivotal role in coronavirus entry into the host cells^5,29,32,33^. Research on the receptor binding domain (RBD) of spike protein affinity to ACE2 can therefore shed some light on how to deal with the coronavirus pandemic. Two forms of spike trimer have been reported in previous studies: open (Apo) and Locked S conformer. Recent findings indicated that switching Apo to Locked form could significantly reduce the spike protein affinity to ACE2 though the interaction of ligands to fatty acid binding pocket (FAB) of spike trimer^34,35^. Protease is an essential enzyme present in viruses^36^. Coronavirus 3-chymotrypsin-like protease (3CLpro), also known as Mpro, is the main protease required for coronavirus proteolytic maturation^36^. Protease enzyme catalyzes proteolytic reactions by cleaving covalent chemical bonds into proteins. It consists of three domains “I (residue 8–101), II (residue 102–184), and III (residue 201–306)” and one loop (residue 185–200: between domain I and II) in which the binding active site of protease located in the cleft between domain I and domain II. Catalytic dyad residues “His41 (domain I) and Cys145 (domain II)”, are located at the active site and play an important role in the catalytic activity of protease^37,38^. Coronavirus protease is considered as a target for several endogenous and exogenous inhibitors^36,39^. In fact, inhibition of protease is one of the most effective ways to treat coronaviral diseases^36,39^. The main phase of SARS CoV-2 main protease has been classified into two states (monomer and homodimer), which are in equilibrium^40^. However, the active form corresponds to homodimer form, according to kinetic studies^40,41^. With regards to the binding sites of the main protease, two allosteric binding sites have been identified experimentally. The first allosteric site is in dimerization domain that including Ile213, Leu253, Gln256, Val297 and Cys300 from protomer A and Tyr118, Asn142 and Cys145 from protomer B and also the second is located in the cleft between main binding site (catalytic domain) and dimerization domain, including mainly these residues: Gln110, Asp153, Val202, Ile249, Pro293, Phe294, and Arg298^42,43^.

In this study, we hypothesized that endogenous steroids (E2, P4, T), exogenous molecules (DEX, H, P) and calcitriol (1, 25-dihydroxyvitamin D3, metabolite or active form of vitamin D) could play a pivotal role in reducing the affinity of coronavirus spike protein to ACE2 and the function of the protease as an inhibitory function. Computational approaches were used to establish the extensive molecular-level interaction of coronavirus spike protein with ACE2 in the presence of steroid structures.

## Methods

### Design of the computation study

#### Phase I: Docking simulation of steroid molecules and coronavirus proteins

Molecular docking was used to detect the precise molecular-level mechanism for the interaction of steroid molecules with coronavirus spike protein and protease. Using docking simulation programs, researchers are able to estimate the interaction energy between ligands and receptors, predict the interaction sites, and classify appropriate ligands conformers^44–46^. The biological activity of the protein depends significantly on its three-dimensional structure under physiological or pathophysiological conditions^47,48^. In fact, the biological activity of proteins can be impaired by blocking their binding sites or active sites of enzymes by toxins, endogenous and exogenous molecules, hormones, medicines, etc^48–50^. Therefore, we intended to conduct research on the potential affinity of male and female sex steroid hormones and DEX, H, P and calcitriol to coronavirus protease and spike protein.

**Figure 1:**
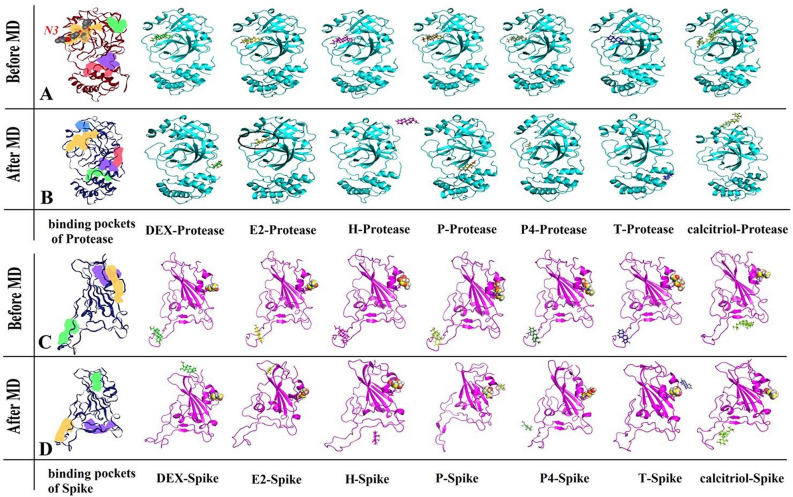
The three-dimensional geometry of the ligand-receptor complex. Best docking geometries (before the MD simulation phase) of steroid molecules on the active site of protease (**A**) or on the contacting sites of the spike protein docking with ACE2 (**C**) and final MD simulation snapshots of steroid molecules on the active site of protease (**B**) or the contacting sites of spike protein with ACE2 (**D**) are shown. Different potential binding pockets of protease and spike protein are shown by yellow (first pocket), violet (second pocket), green (third pocket), red (fourth pocket), and blue (fifth pocket) colors. The glycan linked to receptor-binding domain of spike is represented by ball. Inhibitor N3 is found at the active site of the protease. DEX, dexamethasone; P4, progesterone, T, testosterone, E2, estradiol; H, hydrocortisone; P, prednisone.

The crystallography structure of coronavirus spike protein (PDB ID: 6LZG) and protease (PDB ID: 6LU7), T, P4, E2, DEX, P, H and calcitriol was selected for molecular docking simulation using Auto Dock VINA^51^ to predict the favored orientation of steroids for binding and contacting sites of proteins, as the initial structures for MD simulation^52^. In this study, steroid structures without any rotatable bond were considered to be ligands docked with coronavirus spike glycoprotein (N-acetyl-D-glucosamine linked to the receptor-binding domain at position N343) and protease. After consideration of appropriate Gasteiger and Kollman charges and polar hydrogens, ligand and receptor structures in the pdbqt format were prepared for the protease active site (protomer phase) and allosteric binding site^37,42^, the contacting sites between the spike protein and the ACE2 and spike FAB pocket^30,34,35^. Also, the 6ZB5 PDB ID was used for spike FAB binding pocket simulation study. The grid size of main binding site (active site), allosteric binding sites of main protease, and contacting site as well as FAB binding pocket of spike to points along the x and y and z axes with grid spacing of 1 Å were identified. The main binding site/active site (16.5 × 21.0 × 21.0 Å^3^) and allosteric binding site 1 (16.5 × 15.5 × 25.0 Å^3^) and allosteric binding site 2 (22.5 × 25.5 × 25.5 Å^3^) in main protease and spike protein (contacting site (31.5 × 32.25 × 41.25 Å^3^) and FAB binding pocket (19.5 × 15.0 × 16.5 Å^3^) as the receptor grid box, respectively. Initially, the co-crystallized inhibitor N3 structure was re-docked to the native place in the crystal structure of the protease in order to validate our docking procedure, which produced a binding value of − 13.4 kcal/mol and root mean square deviation (RMSD) of 0.74 Å. The superimposition of these conformers is shown in Fig. S1. Remarkable low value for RMSD (lower than 2 Å as a criterion value) indicated that the assignment of parameters was appropriate for our docking procedure in this study^53^. Ligands and protein structures (spike protein and protease) were optimized by MD simulation prior to the molecular docking procedure. The best docking geometries (before MD simulation phase) of all ligands on the protease (active site, and allosteric binding sites) and spike protein (contacting site and FAB pocket) are depicted in Fig. 1 and Fig. S2.

#### Phase II: MD simulation of the protease and the spike protein in the presence of steroids

MD simulations were used to study the impact of steroids on the function of protease and spike protein, separately. Initially, the complex structure of proteins and steroids obtained from the phase I (docking simulation by AutoDock VINA) with the lowest binding free energy were selected as the receptor and ligand complex in turn for MD simulation. To confirm the MD simulation and MM/PBSA results, certain experimental data (IC_50_) from prior research^54^ were compared to the computationally calculated binding free energy in this study, and quantitative correlations between these two parameters (Log IC_50_ and binding free energy) were thoroughly studied. To achieve the aforementioned goal, four compounds having experimental IC_50_ data (extracted from previous literatures^54^) were docked to the 3CL main homodimer protease, and binding free energy was calculated using MD simulation and the MM/PBSA method. The result indicated an appropriate correlation coefficient (R^2^) between our computationally calculated binding free energy and previous obtained experimental Log IC_50_ data (R^2^ = 0.73). The result and final structures are shown in Fig. S3.

#### Phase III: MD simulation of the spike protein-ACE2 complex in the absence and presence of calcitriol

With regards to the absence of calcitriol (calcitriol: the only steroid molecules which could interact with spike contacting site), MD simulation of spike-ACE2 started from the crystallography structure (6LZG). In this case, spike and ACE2 were glycosylated with N-acetyl-D-glucosamine at position N343 in RBD of spike and N53, N90 and N322 in ACE2. Protein–protein docking simulation approach was conducted on the spike protein-ACE2 complex in the presence of calcitriol using the HADDOCK 2.4 web server^55^. The proteins applied to the HADDOCK 2.4 web server were the complex of spike protein with steroid molecules (from phase II MD simulation, spike protein-calcitriol) and optimized ACE2. The active residues at the contacting sites of two proteins are listed as follows: spike protein (Lys417, Tyr449, Leu455, Phe456, Ala475, Gly476, Phe486, Asn487, Tyr489, Gln493, Gly496, Gln498, Thr500, Asn501, Gly502, Tyr505, Gln506) and ACE2 (Gln24, Thr27, Phe28, Lys31, His34, Glu35, Glu37, Asp38, Tyr41, Gln42, Met82, Tyr83, Lys353, Asp355, Arg357)^30,31^. The residues shown in bold are involved in the formation of hydrogen bonds between spike protein and ACE2. Interactive residues prepared by DIMPLOT were provided in Fig. S4A. The results obtained from the docking of spike protein-calcitriol-ACE2 via HADDOCK had 12 clusters and 166 conformers. The average HADDOCK score for the best cluster (top score) was − 122.0 ± 4.1 (a. u.). The portion of electrostatic energy (− 159.6 ± 24.6 kcal/mol) was greater than the Van der Waals energy (− 71.8 ± 3.4 kcal/mol) in the interaction between spike protein and ACE2 in the presence of calcitriol. The MD simulation and the MM/PBSA approach were then used to calculate the binding free energy of coronavirus spike protein to its receptor, ACE2, in the absence or presence of calcitriol^56,57^.

### MD simulations

In this study, all MD simulations for free molecules were performed in four steps of the water box. Some Na^+^ and Cl^−^ ions were added to reach 140 mM ionic strength. In the first step, the entire system was minimized using the steepest descent algorithm and the process included 50,000 cycles without any position restrictions. In the second and third steps, the equilibration process was completed by a 100 ps NVT set of MD followed by a 100 ps NPT set of restrictions of proteins and sex steroid hormones at the 1000 kJ/mol·nm^−2^ harmonic force constant in the NPT phase. In the final step or production step, 100 ns (for optimizing all molecules, protease-steroids and spike-steroids) and 300 ns (for optimizing spike-ACE2 in absence and presence of calcitriol) MD simulations were carried out without any position restraints. The TIP3P water model was used to design the solvation box of molecules with a minimum distance of 1.5 nm between the solute and the box walls. The simulations were performed at a temperature of 300 K with a time step of 2 fs, employing the LINCS algorithm to constrain the lengths of hydrogen-containing bonds^58^, accounting for the periodic boundary condition (PBC) in equilibration and production processes, and employing GROMACS 2020 with CHARMM 27 force field parameters^59,60^. Topology of steroid molecules was obtained from the SwissParam Web site based on the CHARMM force field parameters^61^. CHARMM-GUI was used to model N-glycan of spike and ACE2 glycoproteins^62,63^. The RMSD of the protein backbone was calculated during a 300 ns MD simulation. Furthermore, specific MD simulation analyses such as solvent accessible surface area (SASA), radius of gyration (Rg) and center of mass (COM) were carried out to better understand the simulated system in the absence or presence of calcitriol.

### Ethical approval

All of the original data in this study come from public databases, and none of the authors conducted any experiments with human subjects.

## Results

### Interaction of steroid molecules with coronavirus protease (the main binding site/active site) and spike protein (the contacting site)

The molecular structure of the active site of protease^37^ and the contacting sites between spike protein and ACE2 (interface between spike protein of SARS-CoV-2 (RBD) and ACE2) after molecular dynamics (MD) simulation^30,31^ are shown in Fig. S5.

The three-dimensional geometry of ligands (steroid molecules) with protease (Fig. 1A) or spike protein (Fig. 1C) complexes was obtained from the lowest energy docking coordinates. The energy values obtained from docking simulation for the interaction of steroid molecules with coronavirus protease or spike protein are shown in Table S1. The minimum binding energy was obtained from docking simulations using the Vina docking algorithm of different steroids with coronavirus protease or spike protein. Binding energies for ligands with protease and spike protein were from − 6.8 to − 8.9 kcal/mol and − 6.0 to − 7.4 kcal/mol, respectively..

As shown in Fig. 1, coronavirus protease and spike protein had several binding pockets for interactions with different molecules. The DoGSite Scorer web server was used to identify these potential binding pockets^64,65^. This web server can be used automatically to predict potential binding pocket and sub-pocket sites based on the calculation of some physico-chemical descriptors, such as volume (Å^3^), surface (Å^2^), depth (Å), surface/volume ratio, hydrogen bond donor–acceptor, the ratio of the polar and non-polar amino acids, etc. In addition, the druggability score was determined using the support vector machine (SVM) method as a machine learning technique^64^. In the drug discovery research, the term druggability is mainly used to depict biological targets, such as proteins that are recognized or predicted as distinct binding sites for drugs with a high affinity. The binding of drugs to druggable sites changes the function of the target bio-macromolecules and can lead to a cure for the patient^66–68^. The druggability score is between 0 and 1 while the higher values are the more druggable pockets^64^. Figure 1 shows the predicted binding pockets of protease and spike protein. The DoGSite Scorer predicted 9 pockets for both proteins. The order of pockets (first to fifth) in this figure was based on the vdW volume and druggability score (> 0.50).

The volume and druggability score of the first pocket of the coronavirus protease (protease inhibitor, N3) was 702.3 Å^3^ and 0.77, respectively. The volume and druggability score of the first pocket of the spike protein was 387.9 Å^3^ and 0.78, respectively. It should be noted that following the MD optimization of protein structures, the number of protein pockets and the first pocket volume and druggability score were changed to 14, 726.0 Å^3^, 0.76 for protease and 10, 355.5 Å^3^, 0.65 for spike protein. Similarly, the position of the protein pockets was changed after the MD simulation, especially for the spike protein. Figure 1A,B show the protease and the spike protein after docking steroid ligands into the active site and the contacting site. Figure 1B,D were prepared from the last conformer of MD simulation of steroid ligands with protease and spike protein, respectively. Having been docked to the active site and the contact site of the protease and spike protein, steroid molecules were considered to form possible interaction with coronavirus proteins at the active site and at the contact site.

**Figure 2:**
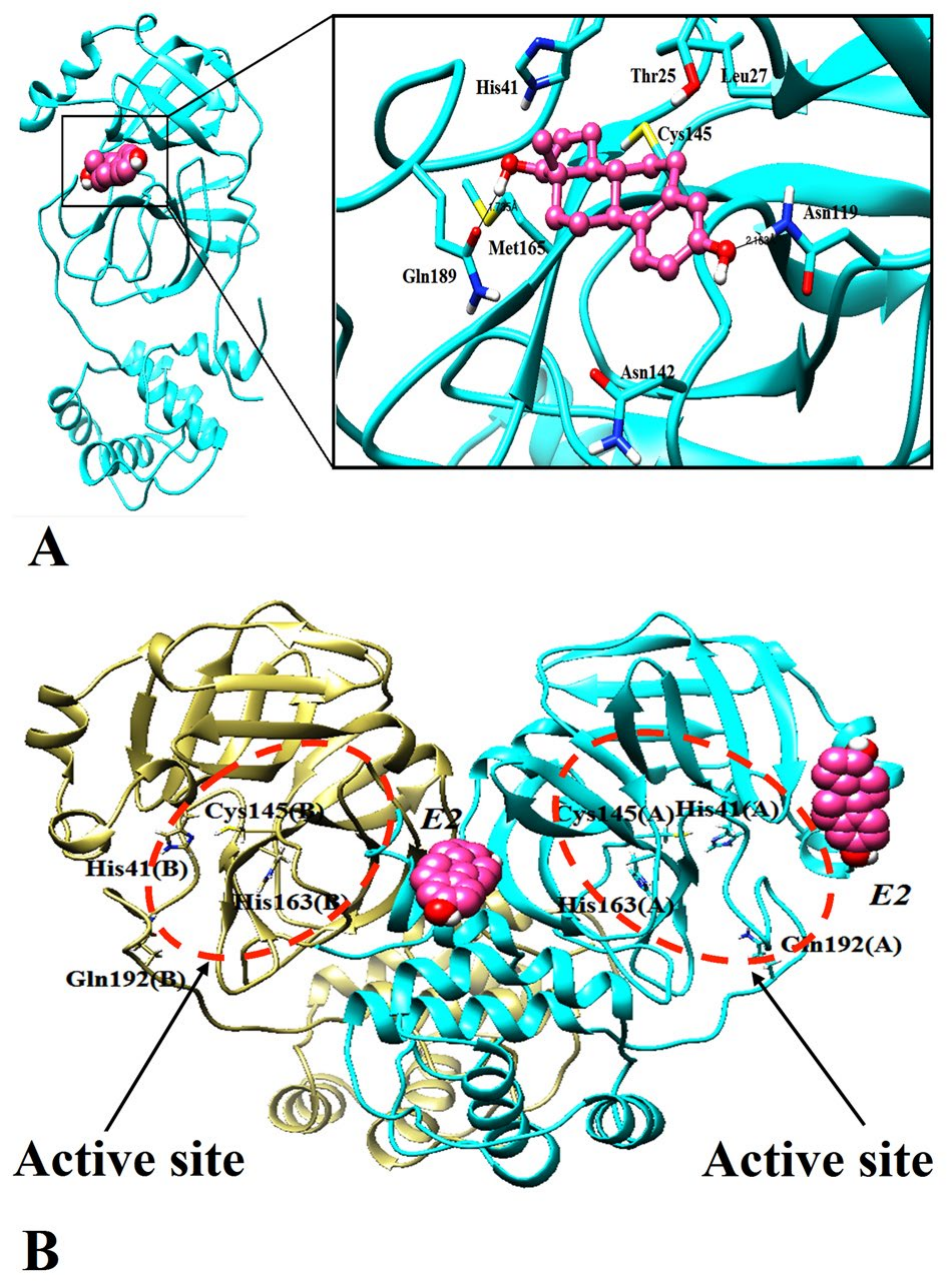
The binding modes of E2 with protease active site. (**A**) The final step of MD simulation of E2 with protease in monomeric state after MD simulation. (**B**) The final step of MD simulation of E2 with homo-dimeric. Formation of hydrogen bonds between E2 and protease residues is shown by black line. Identical residues interacted with E2 depicted by stick form. Protease colored by cyan; ligands indicated by sphere, ball and stick form; E2 is in violet.

**Figure 3:**
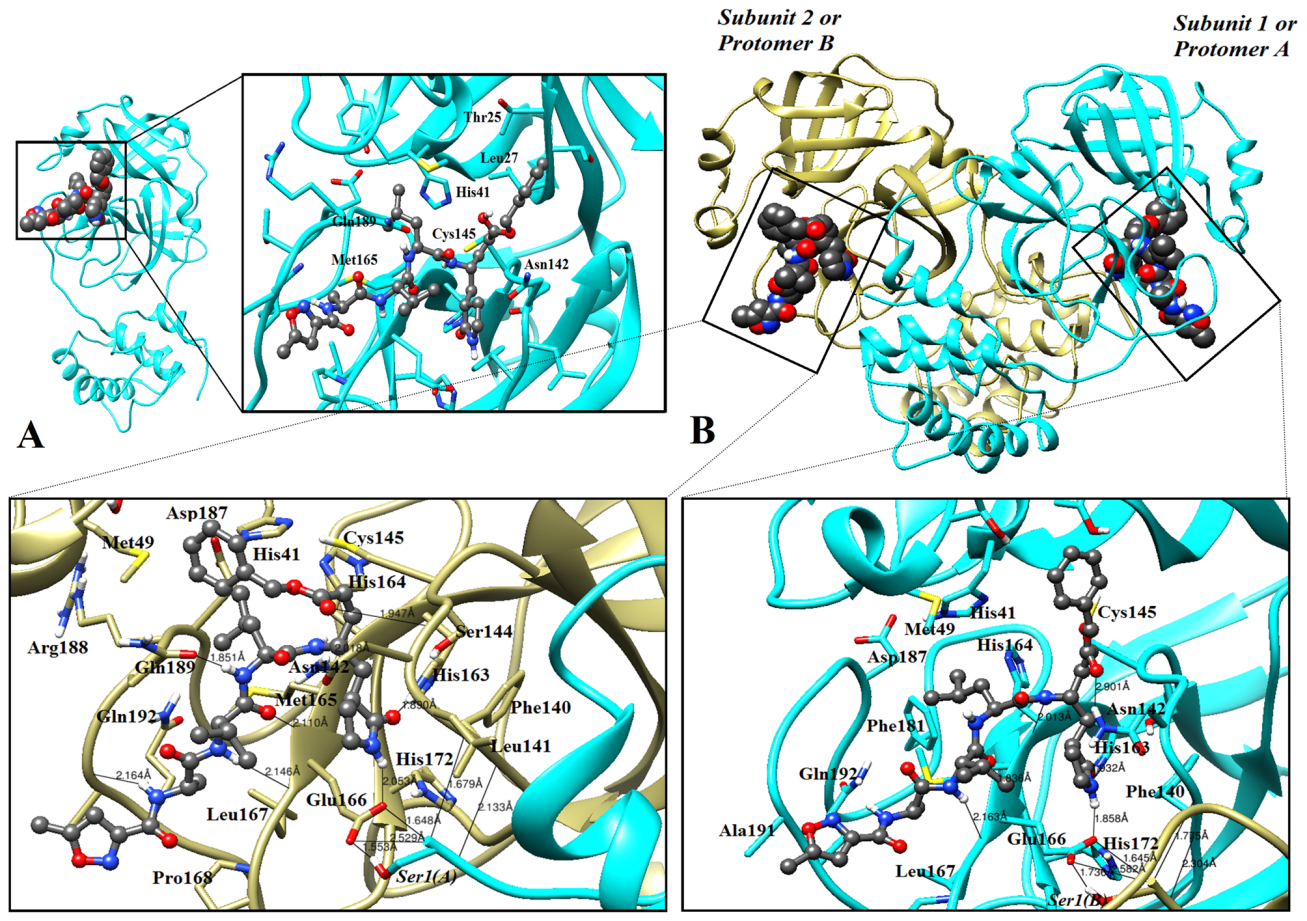
The binding modes of N3 inhibitor with protease active site. (**A**) The final step of MD simulation of Inhibitor N3 in protease active site in monomeric state (crystal structure conformer from 6LU7) after MD simulation. (**B**) The final step of MD simulation of N3 inhibitor with homo-dimeric. Identical residues interacted with inhibitor N3 depicted by stick form. Protease colored by cyan; ligands indicated by sphere, ball and stick form; inhibitor N3 is in grey dark.

As shown inFig. 1B, E2 was the only steroid ligand to occupy the active site of the main protease after 100-ns MD simulation. Interestingly, E2 was able to interact with Thr25, Leu27, His41, Asn142, Cys145, and Met165. E2 formed a hydrogen bond with Gln189 (1.73 Å) and Asn119 (2.16 Å) (Fig. 2A). The potent SARS-CoV-2 protease inhibitor (N3) fits well within the active site of the enzyme^37^. The binding mode of E2 was compared with the inhibitor N3 in the protease active site. The binding mode of the inhibitor N3 was obtained by its crystal structure through protease (PDB ID: 6LU7) after MD simulation (Fig. 3A). It should be noted that ~ 87.5% of the binding residues with protease were shared between E2 and inhibitor N3. E2 interaction and position at the active site of the protease following MD simulation indicated inactivation of protease catalytic function. Unlike E2, the other steroids mainly moved to the other predicted binding pockets. As for the spike protein, calcitriol remained at the contacting site (Fig. 1D). Therefore, we decided to further study the effect of calcitriol on the interaction between spike protein and ACE2.

The effect of steroid molecules on the secondary structure of coronavirus proteins (α-Helix, β-sheet, β Bridge, Turn, coil, Helix 3_10_, and Helix Pi) was studied using the automatic algorithm STRIDE^69^. Figure S6 shows the predicted assignment of secondary structures. As a result, it was predicted that steroids would be potential molecules that could change the conformation of protein secondary structures. For example, the percentage of strands in coronavirus protease was 26.88, which was increased or decreased once steroids interacted with the protease. His41 has been identified as one of the catalytic dyad residues in the protease active site participating in catalytic activity^37^. In the absence of ligands, this residue was associated with the modification of the secondary structure of the protein. However, the interaction of DEX, E2, H, P, and T steroids with the protease has transferred His41 to the Helix 3_10_ structure. As for spike protein, Gln498 is one of the main residues of its contact site that binds to ACE2^30,31^; this residue is found in the Turn structure, and approximately has not been changed by steroids interaction. It should be noted that in the presence of steroids, the percentage of α-Helix, β-sheet, β Bridge, Turn, coil, Helix 3_10_, and Helix Pi in spike protein secondary structure was modified. Overall, these results suggested that the interaction of steroid ligands with protease and spike protein could significantly affect their secondary structure.

### The effect of dimerization of protease on the interaction of Estradiol and N3 inhibitor with the main binding site of protease

**Figure 4:**
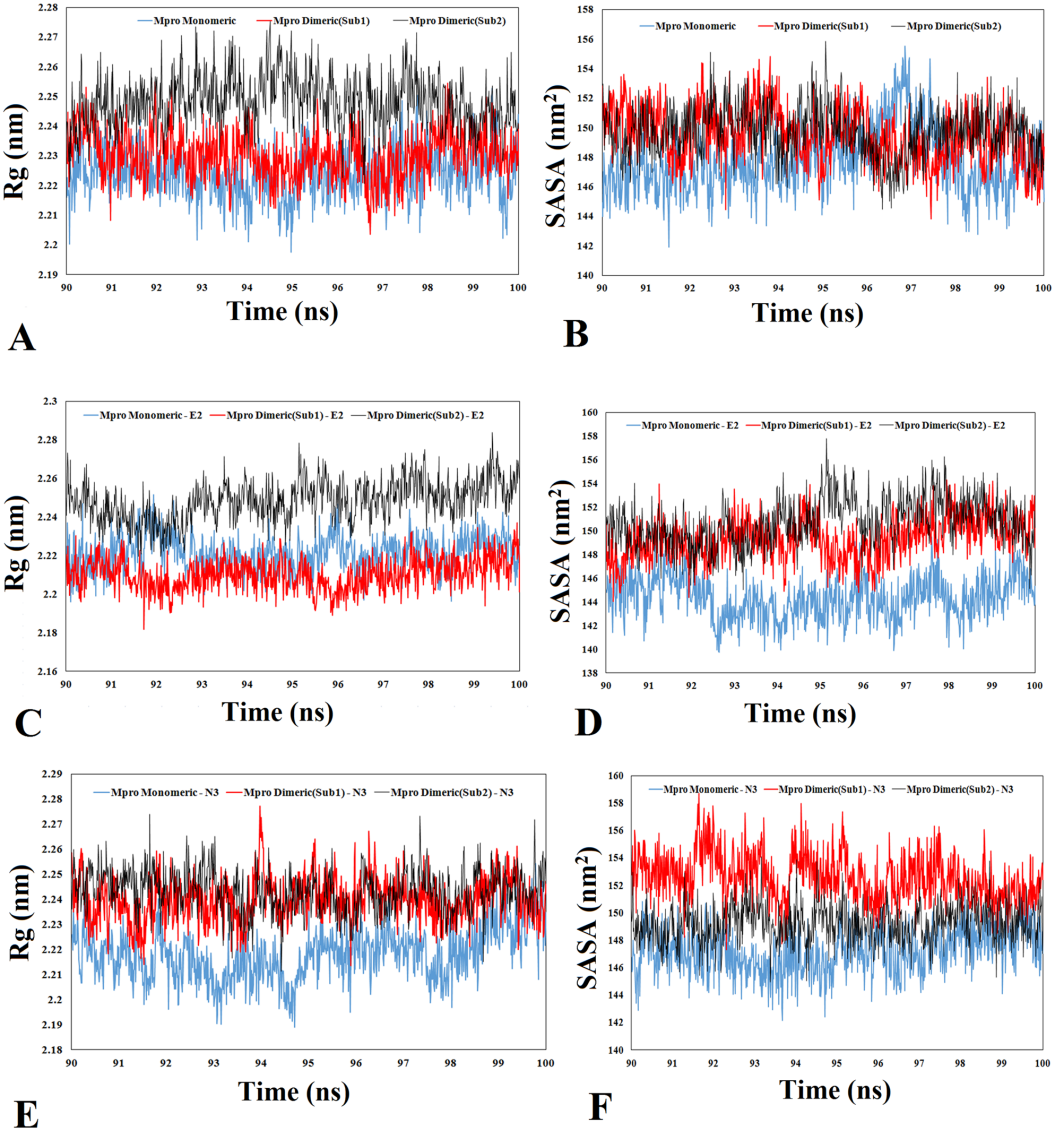
Conformational changes of protease in monomeric and dimeric states. Radius of gyration (RG) and solvent accessibility surface area (SASA) of two forms of protease (**A**, **B**) in absence, and (**C**, **D**, **E**, **F**) presence of E2 and N3 inhibitors.

The interaction of E2 and N3 inhibitor with the two forms of protease (monomeric and dimeric states) has been investigated through docking approaches followed by MD simulation and MM/PBSA methods. The impact of protease dimerization on the interaction of FDA-approved drugs has been studied recently^41^. In fact, the computational data indicated that the best compounds were the potentially inhibitors for the dimeric states (active form)^41^. In this study, N3 inhibitor and E2, which was the only steroid interacting and placing in the main binding site (in the monomeric form), were selected to further study of the effect dimerization on the interaction of ligands. The binding free energy of ligands to protease was calculated by MM/PBSA (Table 1). As shown in Figs. 2 and 3. E2 can only block the main binding site of monomeric state of protease; however, N3 inhibitor can block both forms of protease. Considering the calculated binding free energy, the components of binding free energy are different for monomeric and dimeric forms while interacting with N3 inhibitor. Additionally, the secondary structure analysis indicated that the proportion of proteins secondary structure can change while interacting with ligands in monomeric and dimeric forms (Fig. S7). As an example, in monomeric Mpro-E2, His 41 is in Helix 3_10_, however, in both subunits of dimeric form, this residue is involved in the turn structure. As shown in Fig. S7, the difference in secondary structure in domain I (8–101) is obviously high after transferring from monomeric form to dimeric. These differences are not obvious in domain II and III. To validate the effect of protease folding in two forms of monomeric and dimeric, SASA and Rg analysis were performed (Fig. 4). SASA and Rg analysis indicated that the folding and 3-D structure of protease can be altered while transferring from monomeric to dimeric and vice versa. SASA is the total surface area of proteins that can be accessed by water molecules which was higher in dimeric form of protease in presence of N3 inhibitor and E2. Additionally, the protease undergoes changes in the folding while changing from monomeric to dimeric state and vice versa. The interaction of N3 inhibitor to both subunit of protease in dimeric state is stronger compared to monomeric state (Table 1). However, E2 cannot interact with the main binding site in dimeric state (Fig. 2B). Therefore, it could be concluded that changes occurred in structure of protease in monomeric and dimeric states highly possibly play a pivotal role in interacting with ligands.

**Table 1:** The result of binding free energy calculated by MM/PBSA methods for N3 inhibitor and E2 with the main protease (monomeric and dimeric states) during the last 10 ns of MD simulation.

System	ΔE_vdw_	ΔE_ele_	ΔE_pol,sol_	ΔE_SASA_	Binding free energy (kJ/mol)
M^pro^ Monomeric—N3	− 260.456	− 170.269	269.546	− 28.478	− 189.701
M^pro^ Dimeric_Sub1_—N3	− 228.209	− 209.280	244.435	− 31.686	− 224.765
M^pro^ Dimeric_Sub2_—N3	− 278.260	− 253.965	306.375	− 34.255	− 260.014
M^pro^ Monomeric—E2	− 93.521	− 29.475	72.796	− 11.777	− 62.005
M^pro^ Dimeric_Sub1_—E2	NA	NA	NA	NA	NA
M^pro^ Dimeric_Sub2_—E2	NA	NA	NA	NA	NA

### The possible interaction of steroid to experimentally allosteric binding sites of protease

**Figure 5:**
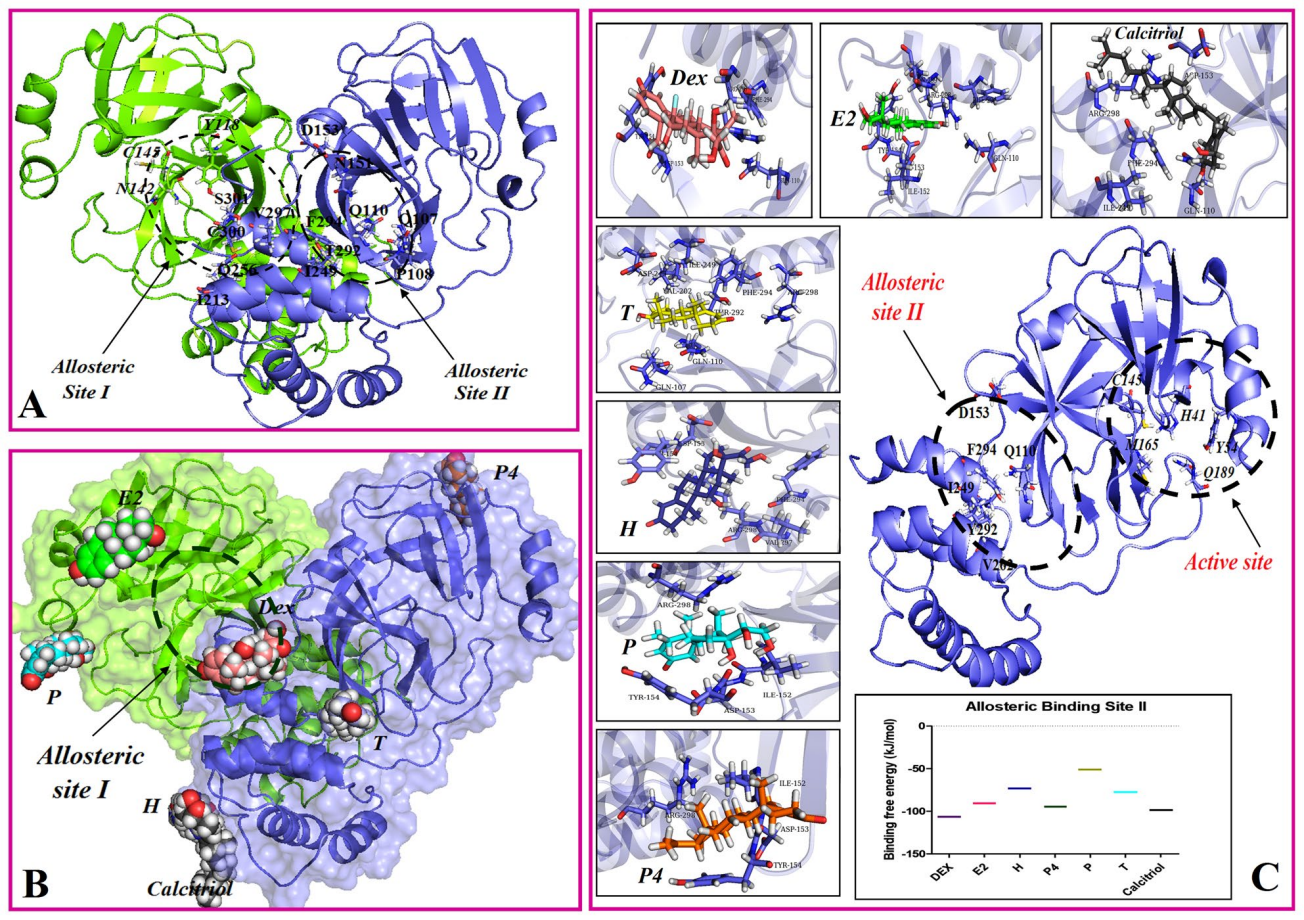
The final snapshot of steroid ligands and first and second experimentally allosteric binding site of the main protease. (**A**) The location of the first (dimerization domain) and second (the cleft between main binding site and dimerization domain) experimentally allosteric binding sites. The final MD simulation snapshots of steroid molecules on the first allosteric site (**B**) and second allosteric site of protease (**C**) are shown. (**C**) The result of binding free energy calculated by MM/PBSA methods for steroid molecules with the second allosteric binding site of protease during the last 10 ns of MD simulation. The obtained data indicated the possible interaction between steroids and the second allosteric sites, however, the first allosteric site cannot be an interaction site for steroids. DEX, dexamethasone; P4, progesterone, T, testosterone, E2, estradiol; H, hydrocortisone; P, prednisone.

The previous literatures identified active site of protease involved in coronavirus proteolytic maturation^36^. In addition to the main binding site, two experimentally allosteric binding sites were identified, which can be the target for allosteric inhibitors^42^. In this study, the possible interaction between steroid ligands and these allosteric binding sites were studied through docking simulation, followed by MD simulation and MM/PBSA analysis. Through performing X-ray crystallographic screen, the location of allosteric inhibitors against protease were recognized: the first and second allosteric sites are located in dimerization domain, and a cleft between the main binding site and dimerization domain, respectively^42^. Our results confirmed the possible interaction between steroids and the second allosteric sites (the cleft), as they could occupy this site during MD simulation time with an appropriate binding free energy (Fig. 5). On the other hand, the data indicated that, the first allosteric site cannot be a target for steroids, as none of them could block this site after 100 ns simulation time. DEX seems to be the most potent ligands (ΔG_bind_ =  − 106.6 kJ/mol), compared to the other steroids.

### The interaction of steroid structure to the fatty acid binding pocket of spike glycoproteins

**Figure 6:**
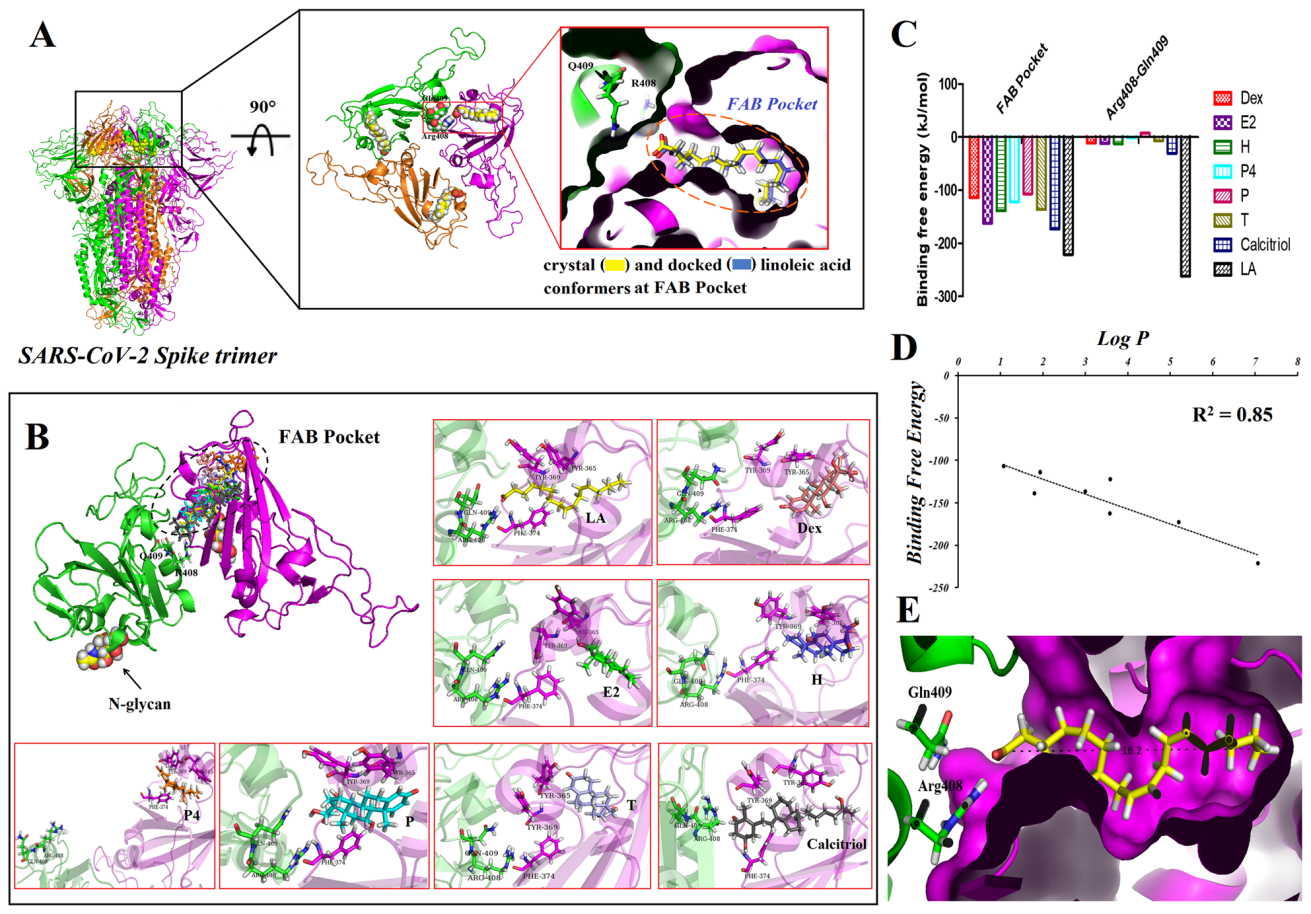
Interacting ligands to the fatty acid binding (FAB) pocket of spike glycoproteins in order to change Apo form to Locked S form. The Linoleic acid (LA) crystal structure (yellow) is superimposed over its docked conformer (blue) (**A**). The possible interaction of steroid molecules to the FAB pocket of spike (**B**). The result of binding free energy calculated by MM/PBSA methods for steroid molecules with the FAB pocket, and Arg408-Gln409 of spike glycoprotein during the last 10 ns of MD simulation (**C**). The correlation between Log P (lipophilicity) and the affinity of ligands to FAB site (**D**). (**E**) The length of FAB pocket (16.2 Å) which is occupied by ligands. DEX, dexamethasone; P4, progesterone, T, testosterone, E2, estradiol; H, hydrocortisone; P, prednisone.

Possible interaction of steroid to the FAB pocket of dimer spike glycoprotein were investigated using docking approaches, followed by MD simulation and MM-PBSA analysis. Our finding indicated that steroid molecules likely interact with FAB pockets. Linoleic acid (LA) is a well-known ligand to interact with FAB pocket and involved in changing Apo form of trimer spike to locked S form through interacting with two sides of trimer spike: firstly by interacting with FAB pocket then secondly by interacting with Arg408 and Gln409 of the adjacent spike monomer (Fig. 6A). Our findings suggested that steroid molecules may interact with FAB pockets. Figure 6B depicts the final steps of the MD simulation trajectories. Furthermore, the interactions of steroid ligands with the adjacent monomer's Arg408 and Gln409 were calculated (Fig. 6C). In this study, the binding free energy and Log P (the partition coefficient ratio calculated by ALOGPS 2.1 program) of steroid ligands and LA were investigated^70^. The data showed that there was a strong correlation between Log P and affinity of ligands to FAB site (Fig. 6D). Compared with steroid ligands, LA had the highest lipophilicity (Log *P* > 7) and affinity to FAB pocket. Calcitriol and E2 which interacted stronger to FAB pocket, had the highest lipophilicity (Log *P* = 5.19 and 3.57, respectively). Therefore, lipophilicity was an important parameter that had an impact on steroid/FAB pocket interaction. Interacting ligands to FAB pocket is the first critical step for production of locked S form of spike glycoprotein. However, ligands cannot have influence unless interacting to Arg408 and Gln409 residues of the next spike. Among the steroids, calcitriol had an appropriate interaction with Arg408 and Gln409, compared to the others (Fig. 6C). Therefore, it could be concluded that similar to LA, calcitriol can fulfill two requirements in order to change Apo form to Locked S form. Except calcitriol, the other steroid ligands cannot significantly influence on Locked S form of spike glycoprotein, although they had an appropriate interaction with FAB pocket residues. What is more, the size and shape of FAB pocket should be considered, since the ligands are required to be long enough (~ 16 Å) to occupy the FAB pocket and have an interaction with Arg408 and Gln409 via hydrogen bonding (Fig. 6E).

### Calcitriol likely affects the interaction of spike protein with ACE2

**Figure 7:**
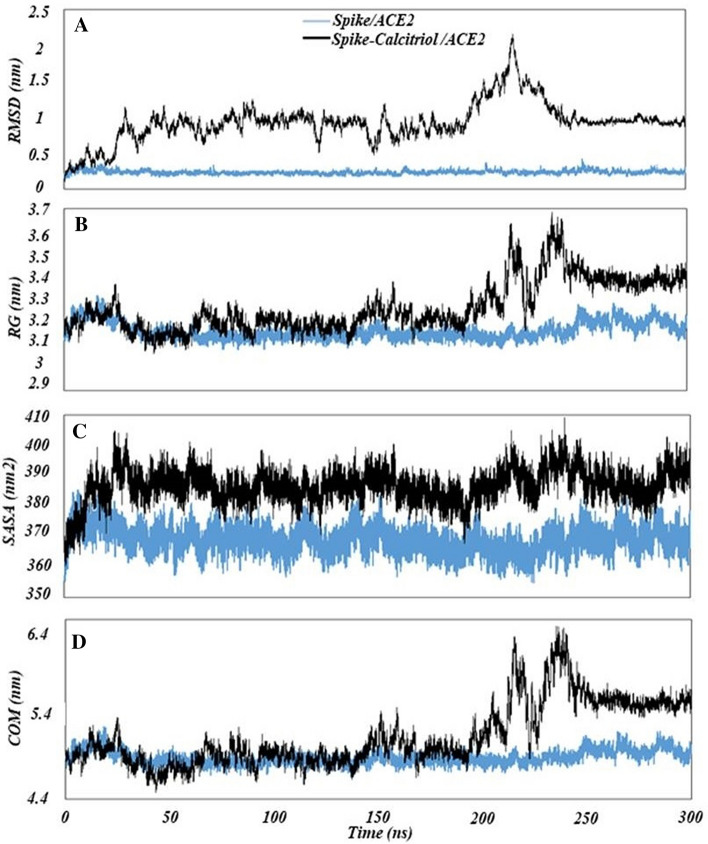
Characteristics of interaction between spike protein and ACE2 following 300 ns MD simulation. (**A**) The RMSD of spike-ACE2 complexes in the absence or presence of ligands. (**B**) The gyration radius (Rg) of spike-ACE2 complexes. (**C**) SASA calculated for the spike protein and ACE2 in basal interactions and in the presence of steroid molecules. (**D**) The center of mass (COM) distances changes between the spike protein and ACE2.

In order to study the effect of steroid molecules (calcitriol) on the interaction between spike protein and ACE2, a MD simulation of 300 ns was performed. The backbone RMSD of proteins was calculated during the MD simulation to ensure that all systems were in an equilibrium state after 300 ns of simulation time (Fig. 7A). The binding free energy and the average number of hydrogen bonds (H-bonds) found between the spike protein and ACE2 are shown in Table 2. The binding energy of spike protein to ACE2 was obtained from the MM/PBSA methods and the average number of hydrogen bonds (H-bonds) between coronavirus spike protein and ACE2 was estimated during the last 100-ns MD simulation (from 200 to 300 ns). The calculated binding energy between the spike protein and ACE2 was − 2270.55 kJ/mol; indicating a remarkable affinity (Table 2). Calcitriol appeared to be effective ligand in this system due to an increase in binding energy. Compared to the basal interaction (spike protein-ACE2 complex interaction), the interaction of one molecule of calcitriol with spike protein-ACE2 complex increased the energy values by 1297.88 (~ 57.2%) kJ/mol (Table 2). The H-bond analysis verified the important role played by steroid molecules in the interaction between spike protein and ACE2 (Table 2). The average number of H-bonds over the last 100 ns of MD simulation showed a sharp decrease in the interaction of one calcitriol (~ 76.11%) to spike protein prior to the interaction of spike protein and ACE2.

**Table 2:** Binding free energy and average number of hydrogen bonds in the spike-ACE2 complex in the absence or presence of calcitriol estimated during the last 100-ns MD simulation (from 200 to 300 ns).

Complexes	Binding free energy (kJ/mol)	Hydrogen bond numbers (Average)
(Spike protein-ACE2)	− 2270.55	8.207
(Spike protein-calcitriol)/ACE2	− 972.67	1.96

**Figure 8:**
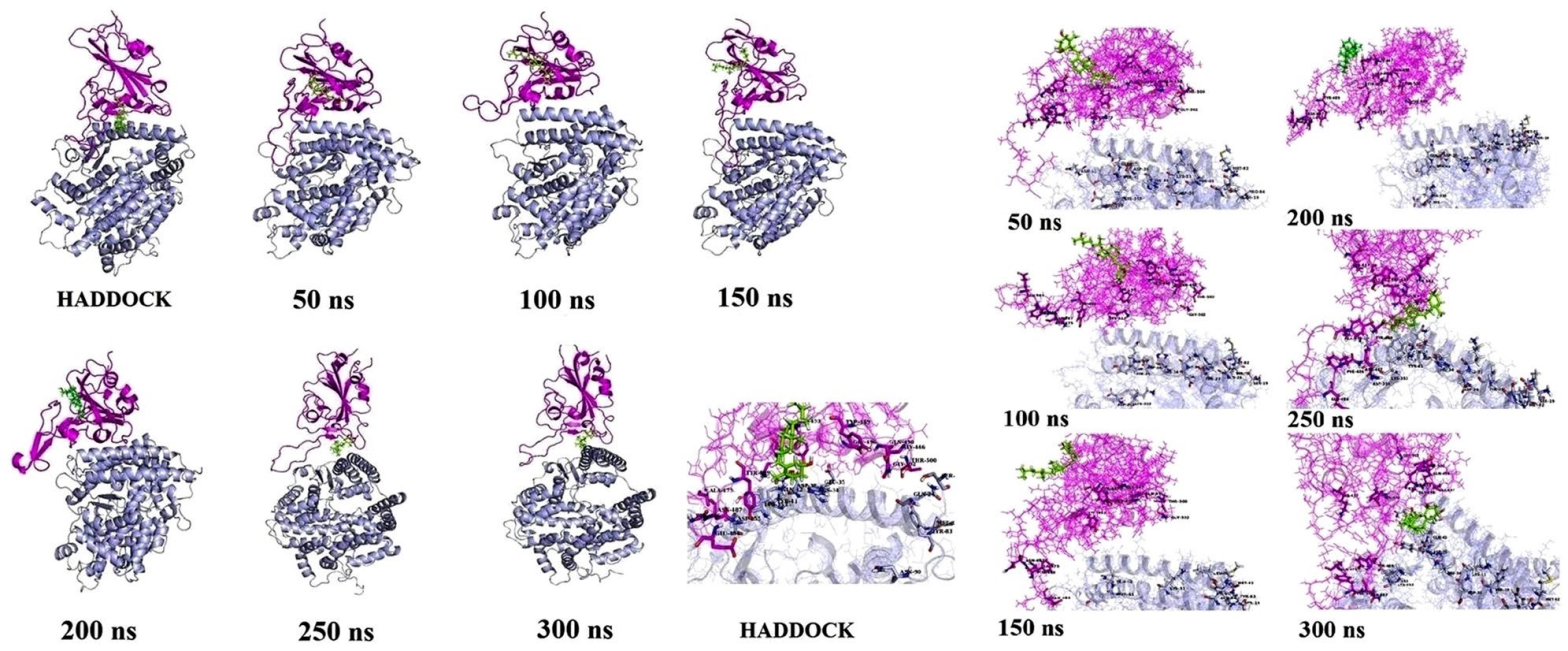
The snapshot was taken from the MD trajectory of the Spike-Calcitriol/ACE2 system at 0, 50, 100, 150, 200, 250, and 300 ns. ACE2, spike, and calcitriol colored by blue, purple and green, respectively.

To investigate the influence of calcitriol on the interaction of spike with ACE2, seven snapshots were collected from the MD trajectory at 0, 50, 100, 150, 200, 250, and 300 ns (Fig. 8). As can be observed, calcitriol was placed around the contacting location of spike and ACE2 in the first stage (0 ns: structure acquired from HADDOCK docking simulation). As time passed (from 0 to 250 ns), calcitriol migrated around the spike protein, changing its orientation to ACE2. However, after 250 ns, it was eventually able to enter the contacting site of two proteins, and the spike-ACE2 complex became stable. Furthermore, MD simulation analysis, namely RMSD, validated the complex's stability after penetrating calcitriol to the contacting site from 250 to 300 ns MD simulation (Fig. 7A). In terms of RMSD, the extremely low oscillation in the basal system (spike/ACE2) compared to the large oscillation in the spike-calcitriol/ACE2 complex illustrated the role of calcitriol on system stability. However, following calcitriol penetration into the contacting site (250–300 ns), the oscillation was substantially reduced, similar to the basal system. As a consequence, the backbone RMSD of proteins (spike protein and ACE2) clearly demonstrated that, due to the close interaction between spike protein and ACE2, the mobility of protein atoms in the spike/ACE2 basal complex was less than that of another complex.

Rg of both spike protein and ACE2 indicated that the docking of calcitriol could make these proteins more unfolded compared to the basal condition, especially the time calcitriol penetrate the contacting site, from 250 to 300 ns (Fig. 7B). To further analyze the changes in the structure of the spike protein-ACE2 complex upon calcitriol binding, SASA of the two proteins during MD simulation was calculated and shown in Fig. 7C. The lowest SASA value was achieved for the spike protein-ACE2 complex compared to another complex; more surface area of the complex was involved in the spike protein/ACE2 complex interaction. The high SASA value in the presence of calcitriol indicated an increase in the number of water molecules covering the protein surface because of increasing the distance between spike and ACE2. To clarify more, the COM distance between spike protein and ACE2, a substantial increase was seen in the space between the two molecules in the presence of calcitriol after 250 ns (Fig. 7D).

Relevant atoms and residues involved in this interaction are shown in Fig. S4B. Based on the DIMPLOT analysis provided by the LigPlot^+^ v1.4.4 software^71^ (presented two-dimensional mode of the interacting residues of spike protein and ACE2 in the absence or presence of calcitriol), the number of effective residues in the spike protein-ACE2 complex at the contacting site was the highest compared to the another system (Fig. S4A). In addition, the type of amino acids involved in binding interactions differed across all systems. As an example, Lys353 ACE2 (chain A) interacted with Gln498 and Gly502 of spike protein (chain B) in the absence of calcitriol influence (formed salt bridge). When calcitriol was docked, some amino acids were not selected as effective amino acids at the contact site compared to the basal complex.

These all findings suggested that the contacting site characteristics of the spike protein were substantially altered in the presence of calcitriol. The interaction of calcitriol with the contacting sites between ACE2 and spike protein remarkably disturbed the formed hydrogen bonds and salt bridges between spike protein and ACE2 residues, suggesting a significant role of this ligand in reducing the affinity between spike protein and ACE2.

## Discussion

The rate of male bias mortality in COVID-19 patients has been documented^72^. Various mechanisms may be responsible for observed sex-biased outcomes of COVID-19 including differences in innate and adaptive immune response^73^ and sex-specific expression of ACE2 as SARS-CoV-2 entry receptor^74^. However, the functions of sex hormones in this sense remain poorly studied. In this study, we provided insights into the interactions of sex steroids with coronavirus spike protein and protease, which could contribute to a shift in coronavirus function and biological activity. We extended our in silico analysis by including other steroids (e.g., calcitriol) and showed that steroid molecules occupied different binding pockets of spike protein and protease surface through a non-covalent interaction. These proteins had a strong tendency to receive steroid molecules, especially, allosteric binding site 2 which show a likely target for steroids. Experimental literatures indicated that the second allosteric site can be a potential binding site for drugs against the main protease of SARS-CoV-2^43^. Considering the active site of protease, E2 was the only steroid structure capable of blocking this site in monomer state. In addition, the active site of proteins shifted when faced with these steroid molecules, suggesting that each molecule had its own binding site. Interestingly, the interaction of calcitriol with the contacting sites of spike protein increased the binding free energy of spike protein-ACE2 interaction. Therefore, calcitriol was more effective in disrupting the binding of spike protein to ACE2 compared to the other steroids. Furthermore, calcitriol highly possibly acts on transforming Apo-form to locked S form, as a potent steroid ligand.

ACE2 is a membrane protein located on the surface of the cells that facilitates the attachment of coronaviruses, such as SARS-CoV, HCoV-NL63 and SARS-CoV-2, to host cell^5,29,33^. An important aspect of infection is the interaction between ACE2 and coronavirus spike protein^5,29,30,32,33^. In fact, this interaction is identified as a critical initial step towards coronavirus penetration, allowing it to pass through the cell membrane^5,29,30,32,33^. Recently, it has been shown that E2 reduces SARS-CoV-2 entry by reducing ACE2 glycosylation^75^. In this study, we found that steroid molecules, such as calcitriol, had a strong binding affinity to the binding site of spike protein, which may disrupt the interaction between the coronavirus spike protein and ACE2. This implied the potential role of calcitriol molecule in reducing the binding of coronavirus spike protein to the ACE2 receptor. DEX, a low-cost steroid drug, is commonly used to decrease inflammation in COVID-19 patients who require ventilation^76^. Recent evidence indicates that a subset of patients with severe COVID-19 may have cytokine storm syndrome^76^ and early administration of short-term corticosteroids improves clinical outcomes for patients with severe COVID-19 pneumonia and evidence of immune hyperreactivity^77^. High dose and short-term corticosteroid therapy at an early stage of respiratory failure have been reported to provide good prognosis for COVID-19 patients^78^. Nonetheless, some clinical evidence does not support corticosteroid therapy for SARS-CoV-2 lung injury and high-dose corticosteroids cannot necessarily be used for the treatment of COVID-19^76^. Further clinical trials are required to clarify the usefulness of steroids for the treatment of COVID-19.

Recent studies have reported variations in the immune response of both men and women to coronavirus infection^1,4–6,12,13^. Officials recorded a 2.8% fatality rate for male patients compared to 1.7% for female patients^79^. However, the mechanism behind this gender gap remains unknown^4,80^. Sex is a significant biological factor to be considered for the prevention and treatment of COVID-19^4,80^. The severity of influenza and other respiratory diseases has been reported to change in response to sex steroid hormones, such as estrogens^81^. Using a simulation analysis, we showed that the E2 was able to bind and block the active site of the main protease. E2 is a predominant sex steroid hormone in the proliferative phase of the ovarian cycle in women. These findings can partly explain the higher resistance of women to SARS-CoV-2.

## Conclusion

In conclusion, our findings suggested that E2 and calcitriol may adversely affect the function of the main protease and the structure of coronavirus spike protein and its interaction with the ACE2 receptor. This may suggest that the use of calcitriol may be more effective in the presence of E2. Therefore, the attachment of coronavirus to ACE2 by administration of calcitriol may be impaired by higher levels of E2 in women during the follicular phase. Moreover, the determination of different phases of ovarian cycle in women infected with COVID-19 should be examined in order to understand the extent of infection and the potential response of female patients to calcitriol. This may also help to determine the need for individual and pathophysiological care of patients. In addition, more research is required to confirm the potential benefits of E2 administration for men with COVID-19. Importantly, cellular activity is changed in response to different physiological or pathophysiological conditions^49^. Therefore, further studies are required to explore the interactive effects of certain physiological or pathophysiological factors on the degree of coronavirus attachment to ACE2 and the severity of infection in response to steroid molecules. These findings expand our understanding of the molecular mechanism of reduced susceptibility of females to COVID-19 which may help to develop new SARS-CoV-2 therapies.

## Supplementary Information

Supplementary Information.

## Data Availability

All data needed to evaluate the conclusions in the paper are present in the paper or the Supplementary Materials.
