# Supplementary material for: The impact of calcitriol and estradiol on the SARS-CoV-2 biological activity: a molecular modeling approach
Source: Sci Rep. 2022 Jan 13;12:717. doi: 10.1038/s41598-022-04778-y (PMC8758694; doi:10.1038/s41598-022-04778-y)
Supplement: Supplementary file 1 — Supplementary Information. [file 41598_2022_4778_MOESM1_ESM.doc]

**The impact of calcitriol and estradiol on the SARS-CoV-2 biological activity: A molecular modeling approach**

Alireza Mansouri^1¶^, Rasoul Kowsar^2,1,*¶^, Mostafa Zakariazadeh^3,4^, Hassan Hakimi^5,6^ and Akio Miyamoto^1*^

^1^ Global Agromedicine Research Center (GAMRC), Obihiro University of Agriculture and Veterinary Medicine, Obihiro, Hokkaido, Japan.

^2^ Department of Animal Sciences, College of Agriculture, Isfahan University of Technology, Isfahan, Iran.

^3^Department of Biology, Payame Noor University, PO BOX 19395-3697, Tehran, Iran

^4^Research Institute of Bioscience and Biotechnology, University of Tabriz, Tabriz, Iran

^5^National Research Center for Protozoan Diseases, Obihiro University of Agriculture and Veterinary Medicine, Obihiro, Hokkaido, Japan

^6^Department of Veterinary Pathobiology, College of Veterinary Medicine, Texas A&M University, College Station, Texas, United States of America

^¶^These authors contributed equally to the manuscript.

***Corresponding authors:** Akio Miyamoto ([akiomiya@obihiro.ac.jp](mailto:akiomiya@obihiro.ac.jp)); Rasoul Kowsar (Rasoul_kowsarzar@yahoo.com)


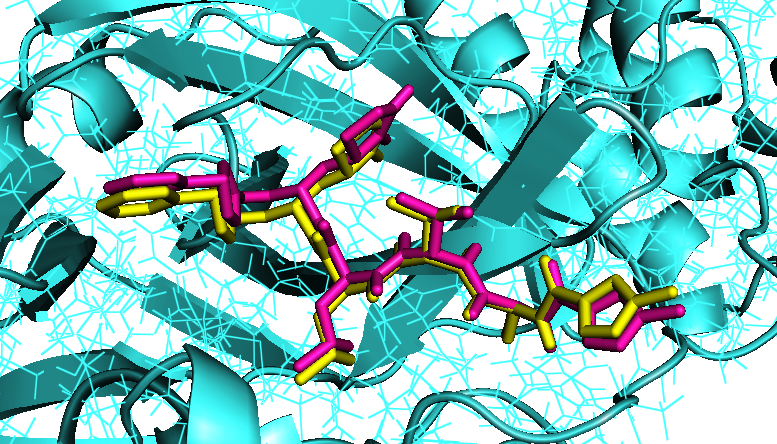


**Fig. S1.** Superimposition of inhibitor N3 crystal structure (yellow) with its docked conformer (violet). Calculated RMSD between two structures is 0.74Å. Protease indicated by cyan color.


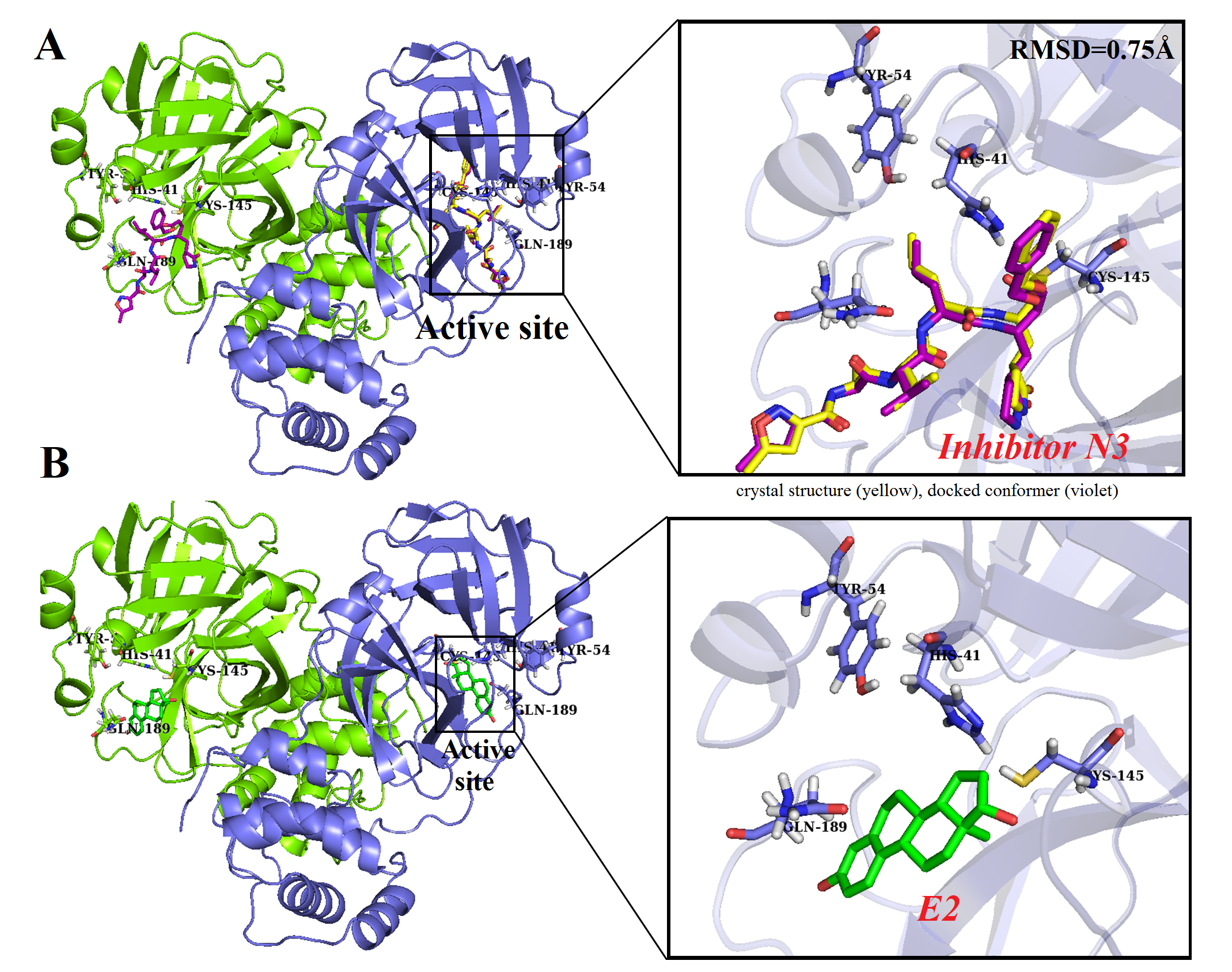


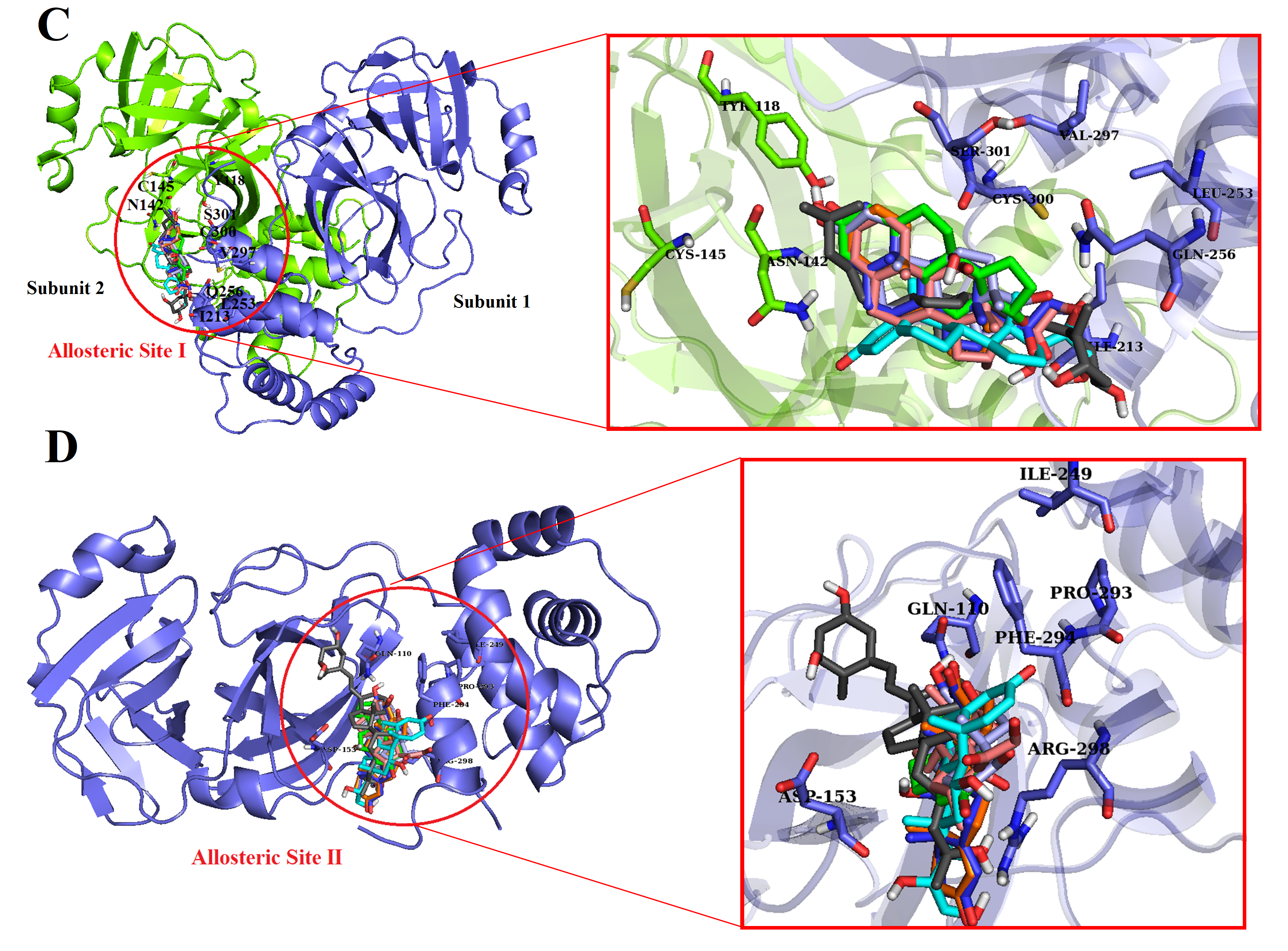


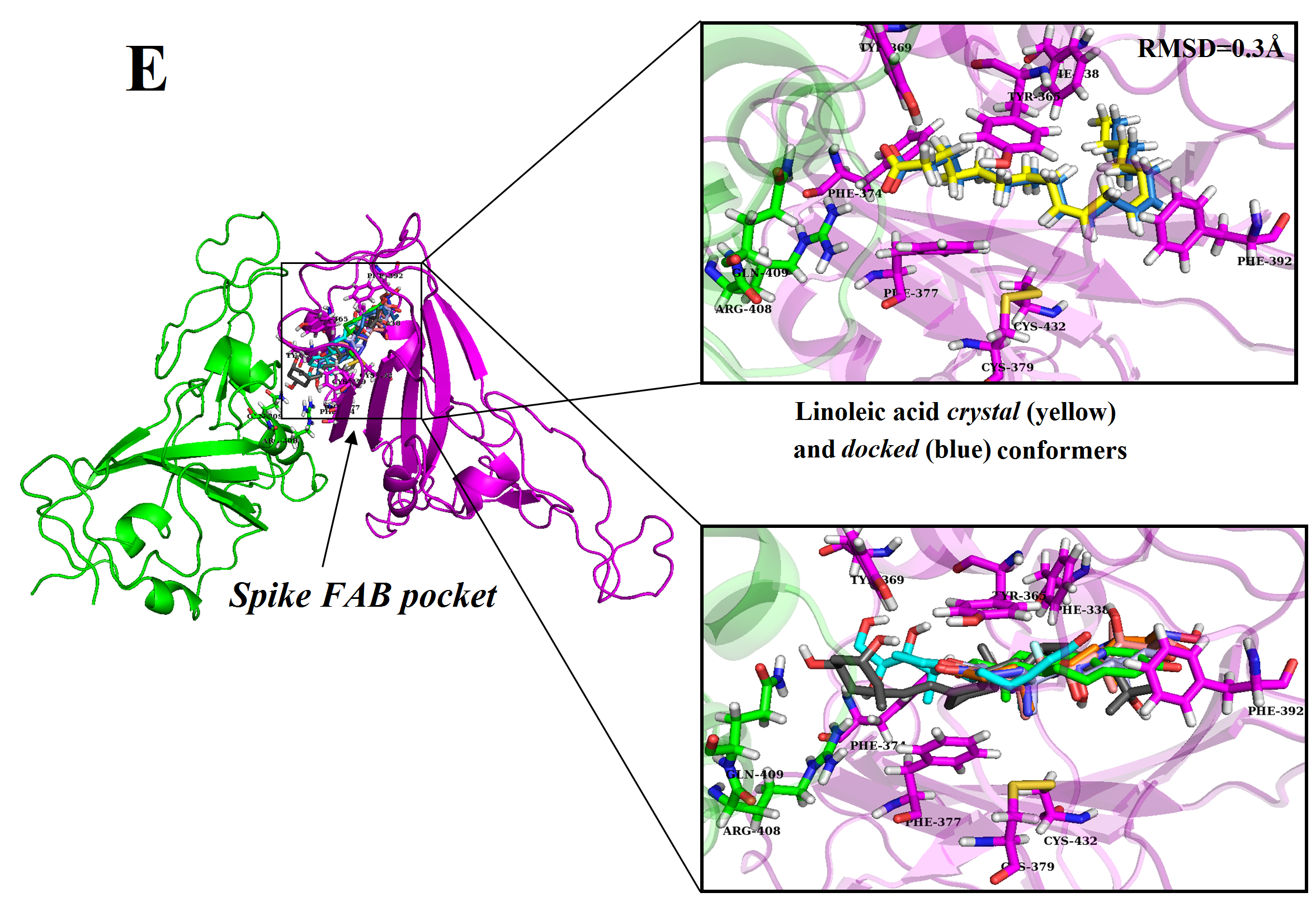


**Fig. S2.** The best docking geometries (before MD simulation phase) of all ligands on the protease and spike protein. (A) N3 inhibitor and active site, (B) E2 and active site, (C) steroid molecules and allosteric binding site1, (D) steroid molecules and allosteric binding site2, (E) streoid molecules and FAB pocket.


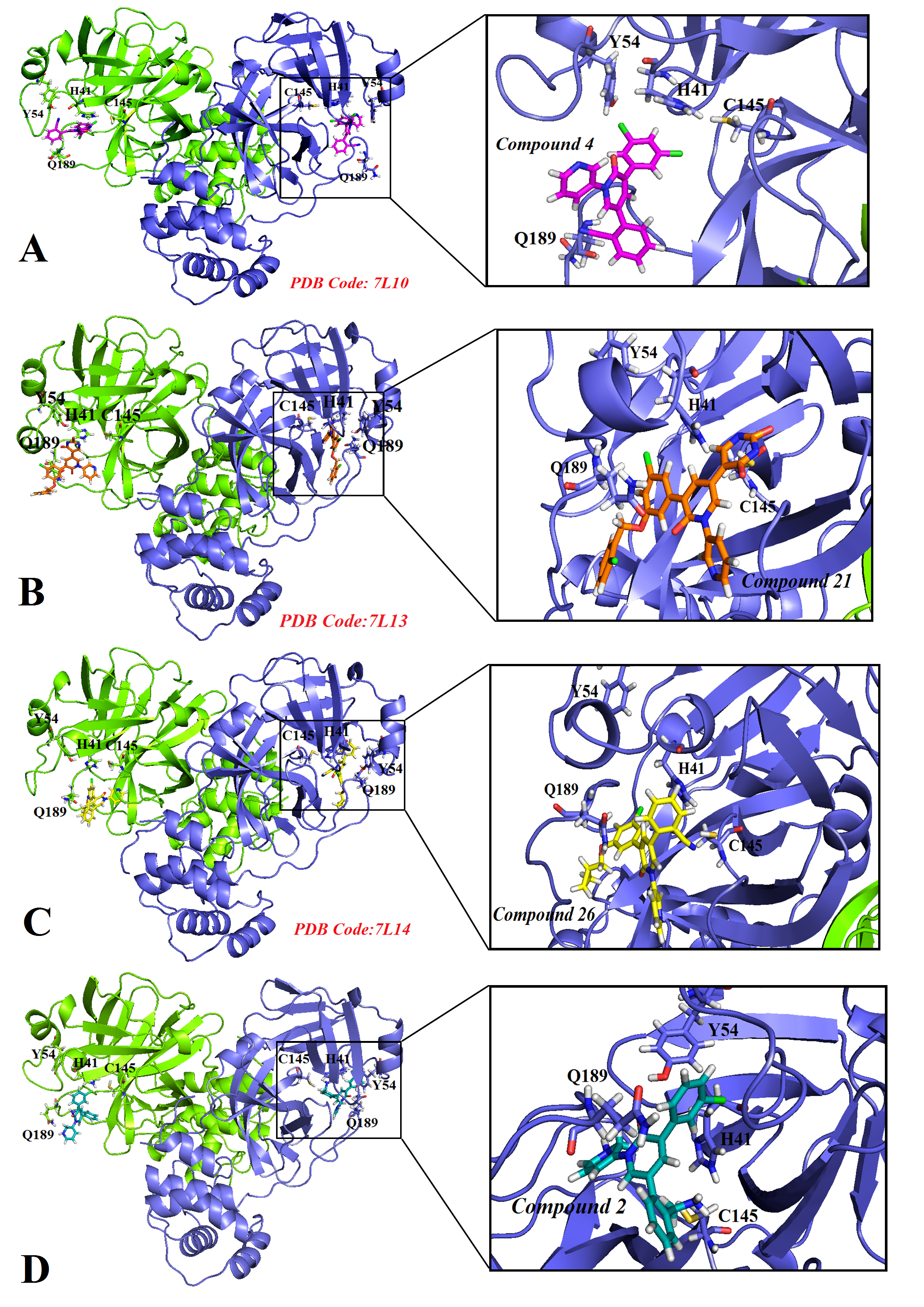


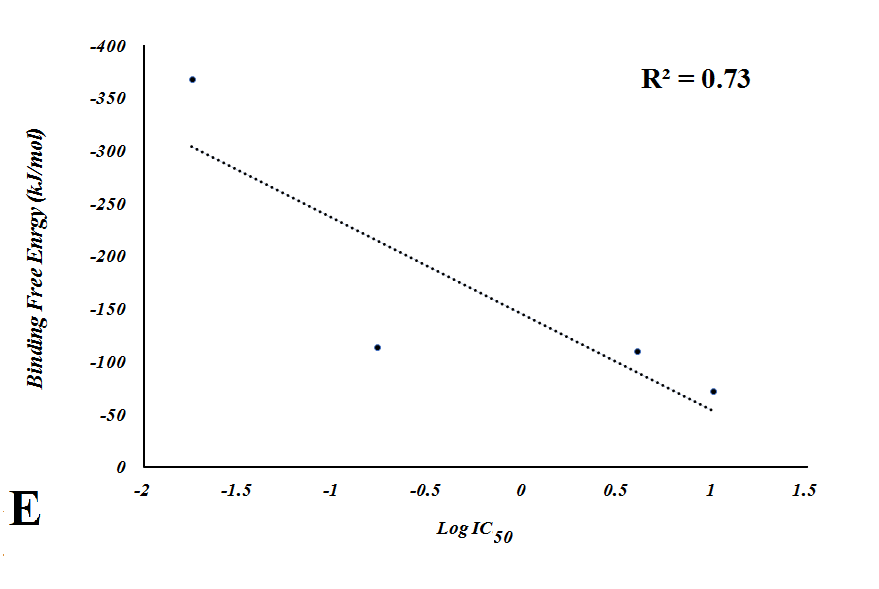


**Fig. S3.** The correlation coefficient (R2) between the computationally calculated binding free energy (MM/PBSA) and experimental Log IC50 data (1). (A) Compound 4 and homo-dimer protease, (B) Compound 21 and homo-dimer protease, (C) Compound 26 and homo-dimer protease, (D) Compound 2 and homo-dimer protease, and (E) R2 between binding free energy and Log IC50.


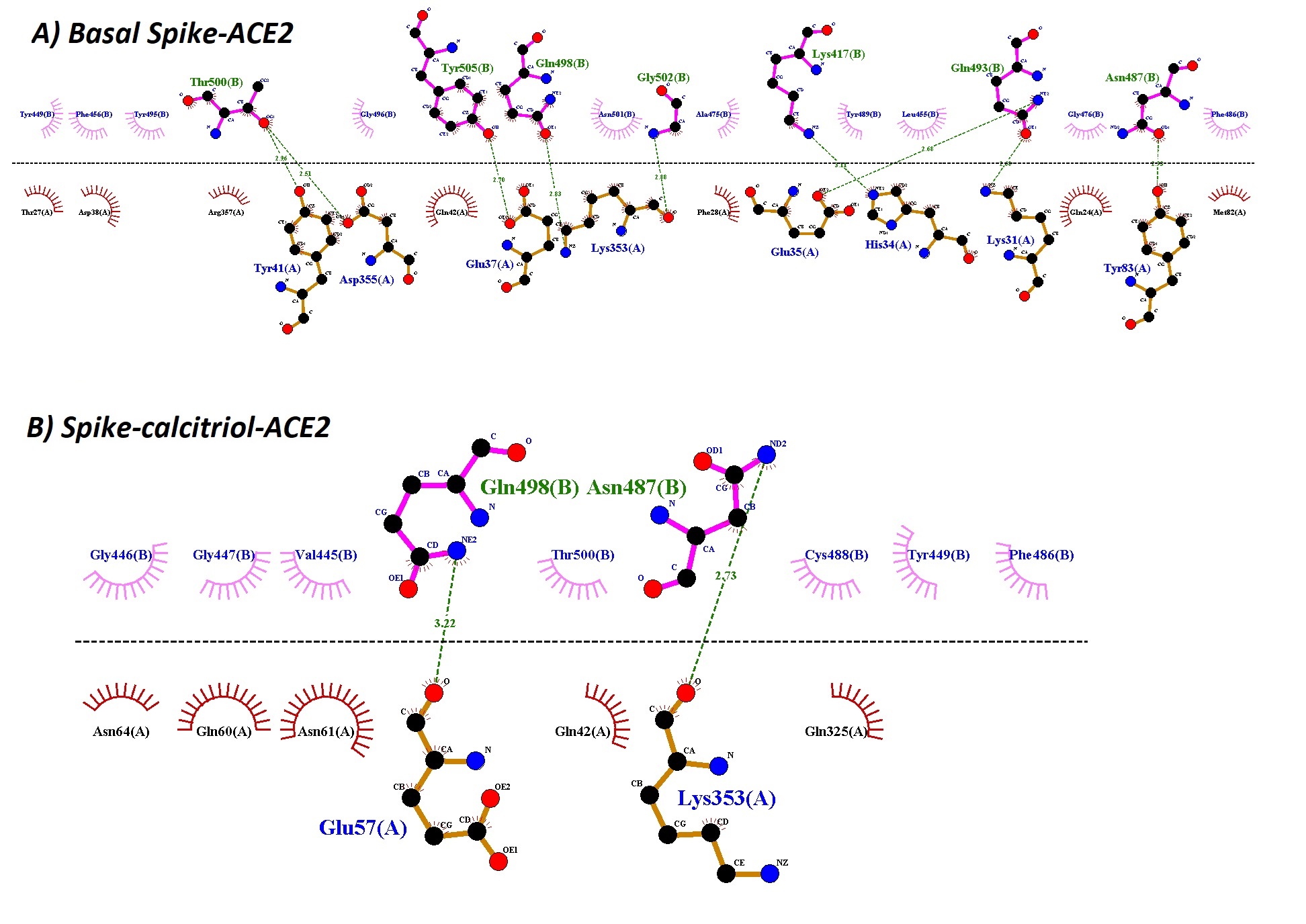


**Fig. S4.** Interacted of spike protein (chain B) with ACE2 (chain A) residues in basal complex state (A) and in the presence of calcitriol (B).


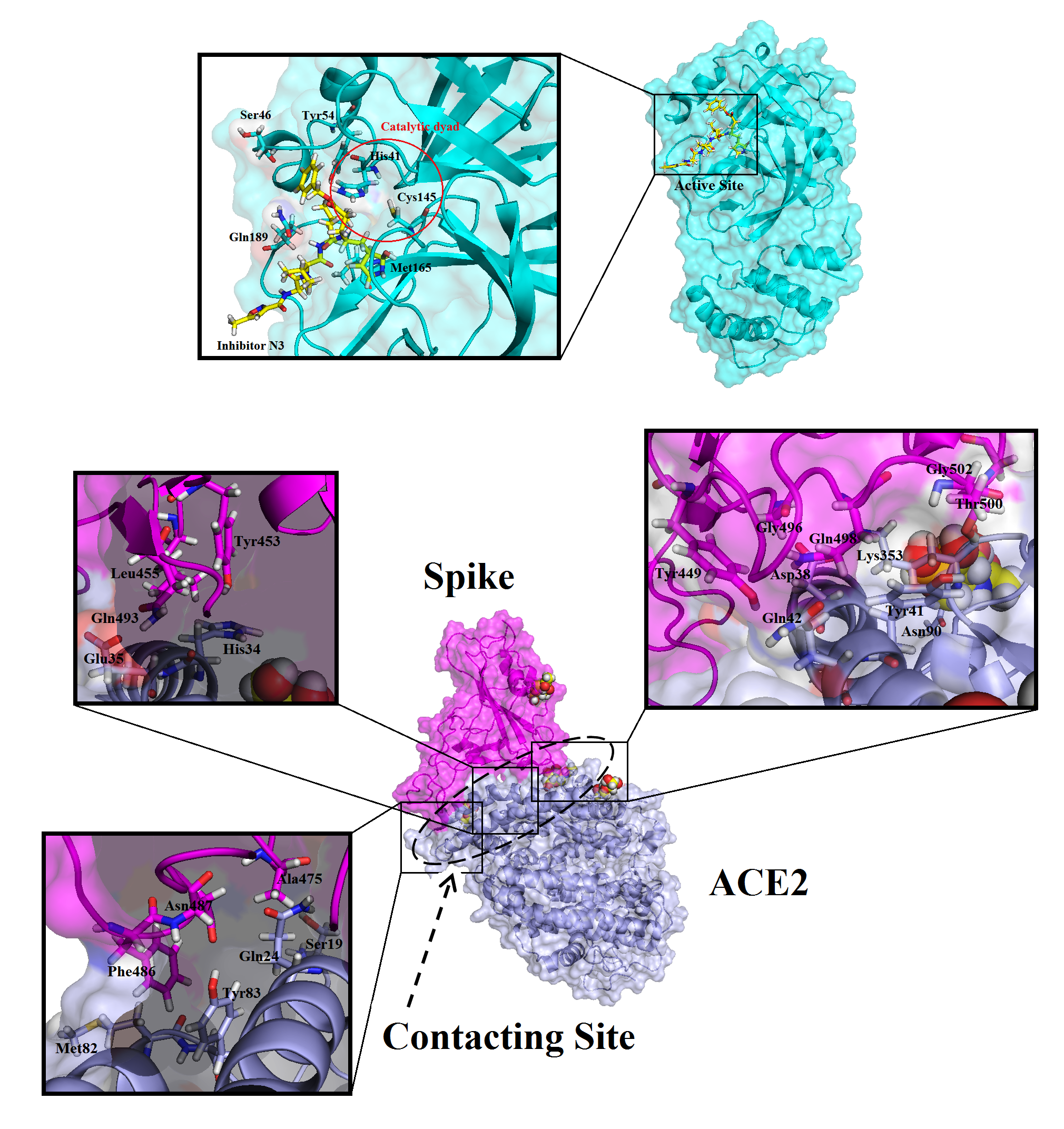


**Fig. S5.** The structures of SARS-CoV-2 protease and spike protein bound to ACE2 after MD simulation. Proteins presented by secondary structure and surface show. The boxed regions are interacting residues of protease binding active site with inhibitor N3 and interacting residues in the interface between spike protein and ACE2 (contacting sites). Residues depicted by stick form.

Glycosylated residues of Asn343 Spike and Asn53, Asn90 and Asn322 ACE2 depicted by sphere form.

**Table S1.** Calculated free binding energy values of seven ligands docked at the main protease or at the spike protein. The unit of energy is Kcal/mol.

|  | Spike | | Protease | | | Homo-dimer Protease | |
| --- | --- | --- | --- | --- | --- | --- | --- |
|  | Contacting site | FAB pocket | Active site | Allosteric  site I | Allosteric site II | Subunit I | Subunit II |
| DEX | -6.8 | -10.9 | -7.2 | -5.5 | -8.8 | ---- | ---- |
| E2 | -6.5 | -10.9 | -6.8 | -5.1 | -7.2 | ---- | ---- |
| H | -6.9 | -10.1 | -7.7 | -5.6 | -9.3 | ---- | ---- |
| P | -7.4 | -8.4 | -8.9 | -6.3 | -8.4 | ---- | ---- |
| P4 | -6.1 | -11.1 | -7.3 | -5.8 | -8.3 | ---- | ---- |
| T | -6.0 | -10.8 | -6.9 | -5.2 | -7.8 | ---- | ---- |
| Calcitriol | -7.3 | -7.7 | -7.7 | -6.2 | -9.3 | ---- | ---- |
| Linoleic acid | ---- | -11.7 | ---- | ---- | ---- | ---- | ---- |
| Inhibitor N3 | ---- | ---- | ---- | ---- | ---- | -11.3 | -11.3 |
| E2 | ---- | ---- | ---- | ---- | ---- | -6.7 | -6.7 |
| *Compound 2* | ---- | ---- | ---- | ---- | ---- | -8.4 | -8.4 |
| *Compound 4* | ---- | ---- | ---- | ---- | ---- | -9.2 | -9.3 |
| *Compound 21* | ---- | ---- | ---- | ---- | ---- | -12.1 | -10.5 |
| *Compound 26* | ---- | ---- | ---- | ---- | ---- | -12.0 | -12.4 |

DEX, dexamethasone; P4, progesterone, T, testosterone, E2, estradiol; H, hydrocortisone; P, prednisone.


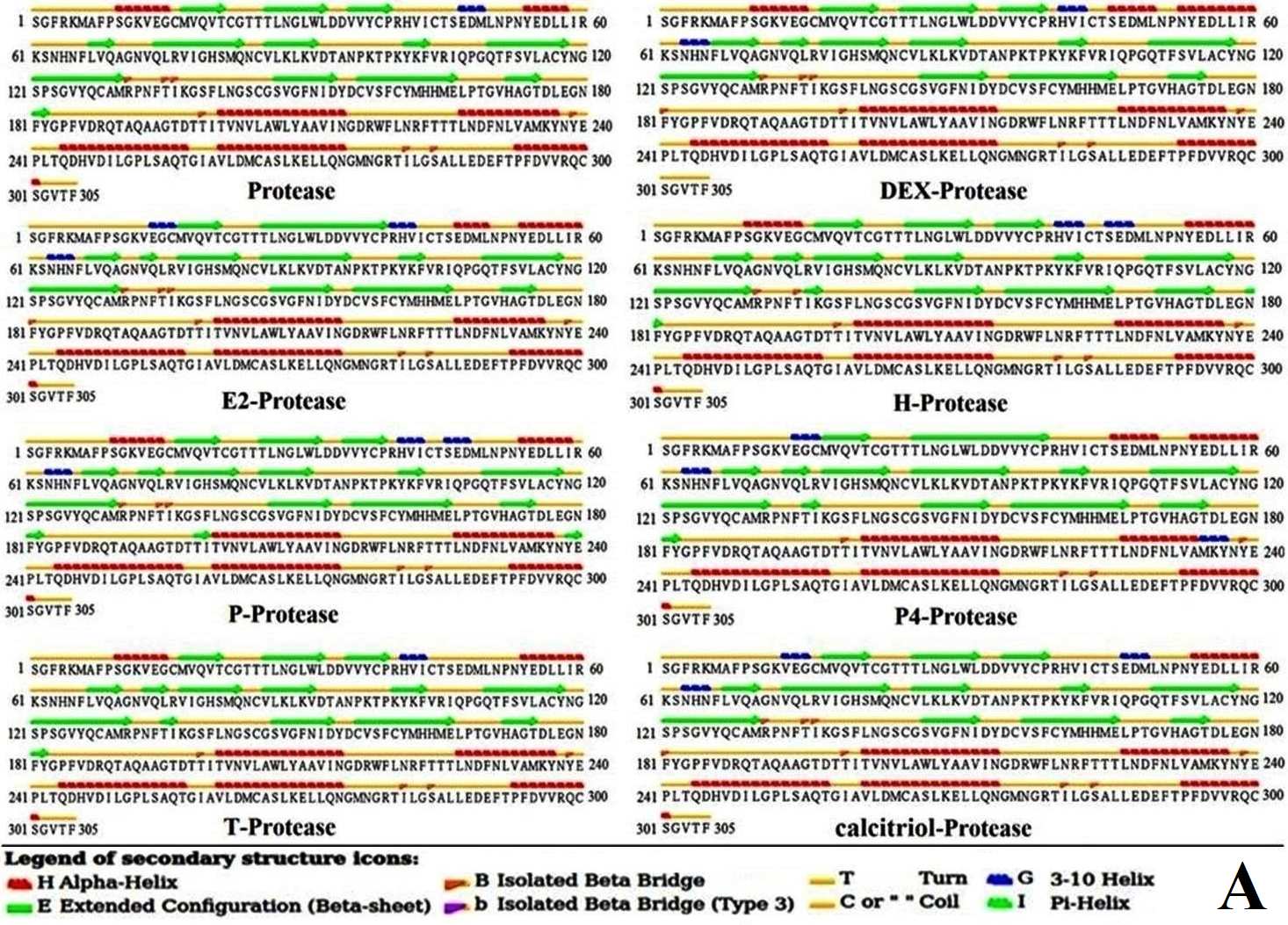


**
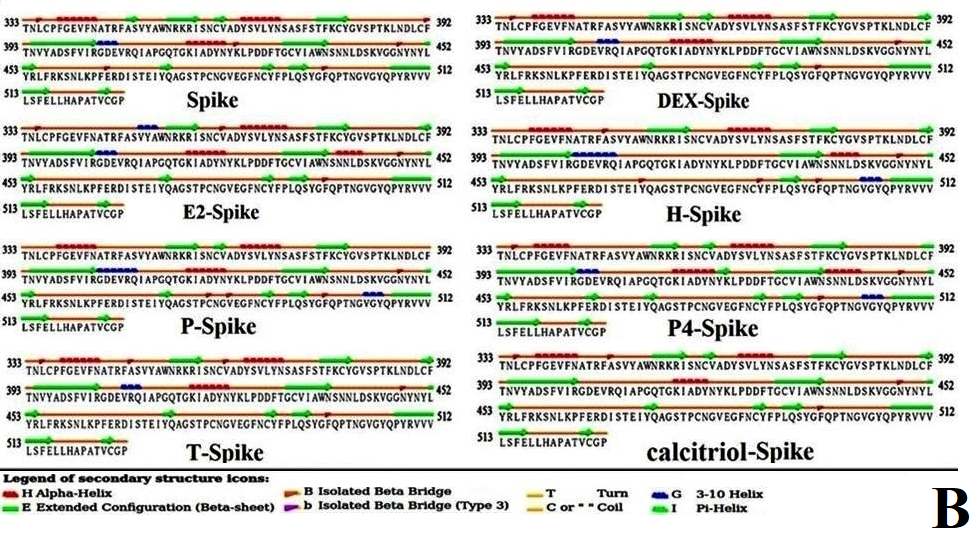
**

**Fig. S6.** Computed secondary structure changes in protease and spike protein in the absence or presence of ligands after 100 ns MD simulation by the STRIDE algorithm. DEX, dexamethasone; P4, progesterone, T, testosterone, E2, estradiol; H, hydrocortisone; P, prednisone.


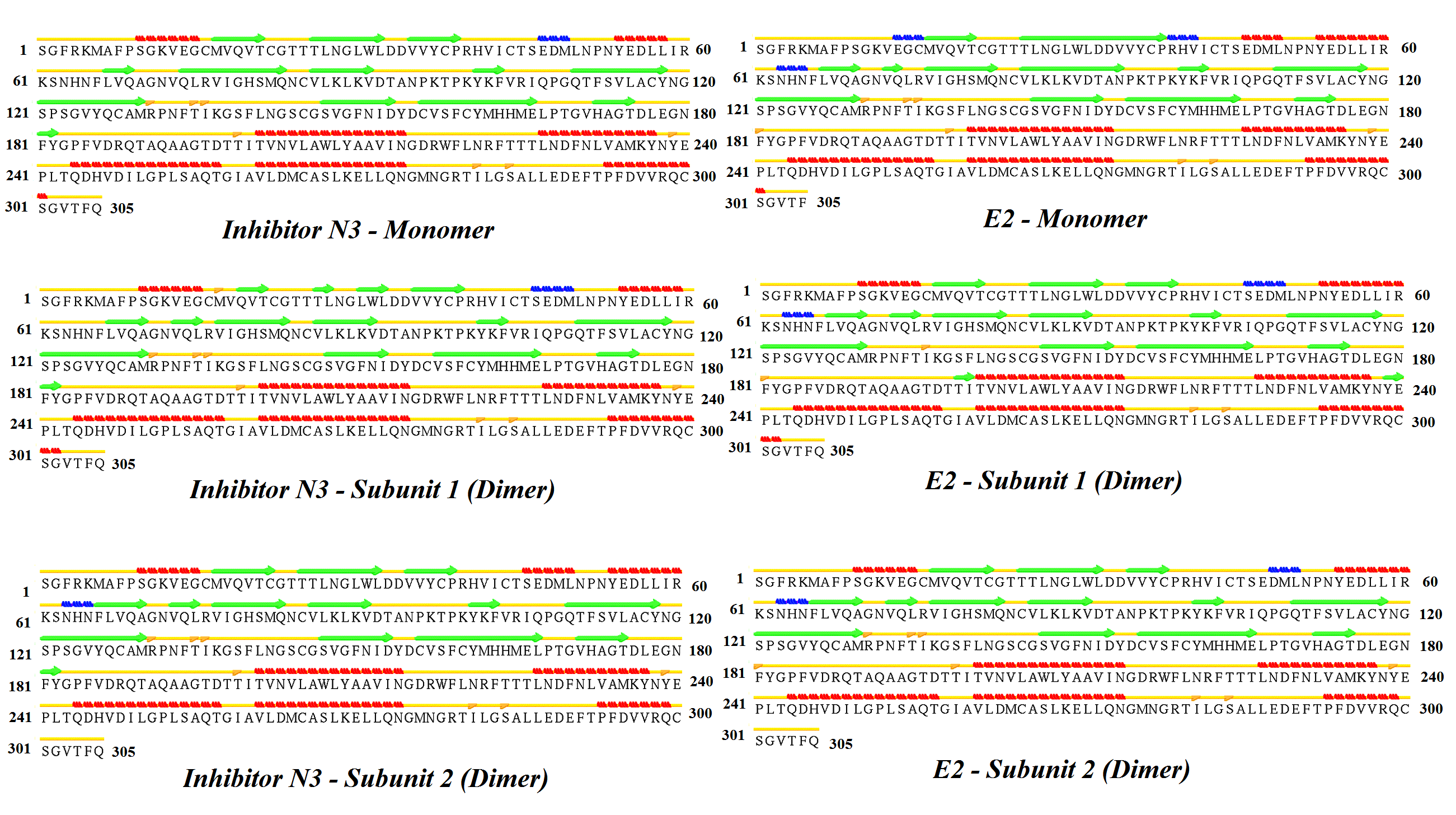


**Fig. S7.** Computed secondary structure changes in monomeric and dimeric protease in the presence of N3 inhibitor and E2 after 100 ns MD simulation by the STRIDE algorithm.

**References**

1. Zhang, C.-H. *et al.* Potent noncovalent inhibitors of the main protease of SARS-CoV-2 from molecular sculpting of the drug perampanel guided by free energy perturbation calculations. *ACS Cent. Sci* **7**, 467-475 (2021).
